# Psychopharmacological Therapy Positively Modulates Disease Activity in Inflammatory Bowel Disease: A Systematic Review

**DOI:** 10.3390/ijms26136514

**Published:** 2025-07-06

**Authors:** Federica Di Vincenzo, Antonio Maria D’Onofrio, Angelo Del Gaudio, Elena Chiera, Gaspare Filippo Ferrajoli, Francesco Pesaresi, Alessio Simonetti, Marianna Mazza, Georgios Demetrios Kotzalidis, Mauro Pettorruso, Giovanni Martinotti, Loris Riccardo Lopetuso, Antonio Gasbarrini, Gabriele Sani, Gionata Fiorino, Franco Scaldaferri, Giovanni Camardese

**Affiliations:** 1 IBD Unit, Digestive Disease center (CeMAD), Department of Translational Medicine and Surgery, Fondazione Policlinico Universitario Agostino Gemelli IRCCS, 00168 Rome, Italy; 2 Università Cattolica del Sacro Cuore, 00168 Rome, Italy; 3 Department of Neuroscience, Section of Psychiatry, Università Cattolica del Sacro Cuore, 00168 Rome, Italy; 4 Dipartimento di Salute Mentale, Ospedale di Latina, ASL Latina, 04100 Latina, Italy; 5 Department of Neuroscience, Section of Psychiatry, Fondazione Policlinico Universitario Agostino Gemelli IRCCS, 00168 Rome, Italy; alessio.simo@gmail.com (A.S.); marianna.mazza@policlinicogemelli.it (M.M.); g.camardese@unilink.it (G.C.); 6 Menninger Department of Psychiatry and Behavioral Sciences, Baylor College of Medicine, Houston, TX 77030, USA; 7 Department of Neuroscience, Imaging and Clinical Science, University “G. D’Annunzio” Chieti-Pescara, Via Luigi Polacchi, 11, 66103 Chieti, Italy; 8 Department of Life Science, Health, and Health Professions, Link Campus University, 00165 Rome, Italy; 9 Internal Medicine, Division of Internal Medicine and Gastroenterology, Fondazione Policlinico Universitario Agostino Gemelli IRCCS, Università Cattolica del Sacro Cuore, 00168 Rome, Italy; 10 CeMAD Translational Research Laboratories, Digestive Disease Center (CeMAD), Department of Medical and Surgical Sciences, Fondazione Policlinico Universitario Agostino Gemelli IRCCS, 00168 Rome, Italy; 11 IBD Unit, Department of Gastroenterology and Digestive Endoscopy, San Camillo-Forlanini Hospital, 00152 Rome, Italy

**Keywords:** inflammatory bowel disease, psychiatric medications, gut–brain axis, antidepressants, disease activity

## Abstract

Depression, anxiety, and perceived stress are common comorbidities in patients with inflammatory bowel disease (IBD) and may negatively influence the disease course. Likewise, severe IBD may contribute to the development or worsening of psychiatric symptoms. Despite the established relevance of the gut–brain axis and frequent use of psychotropic medications in IBD patients, limited evidence exists regarding the effects of psychiatric treatments on gastrointestinal disease activity. Therefore, the aim of this systematic review is to evaluate the effectiveness of psychiatric therapies on gastrointestinal symptoms and disease activity in patients with IBD. The work was conducted in accordance with PRISMA guidelines. Searches were performed across PubMed, Web of Science, and Scopus up to July 2024. Eligible studies evaluated the effectiveness of psychiatric medications—including antidepressants, antipsychotics, anxiolytics, sedative-hypnotics, mood stabilizers, anticonvulsants, and others—on at least one gastrointestinal outcome in patients with IBD. Outcomes included changes in commonly used clinical and endoscopic scores for Crohn’s disease (CD) and ulcerative colitis (UC), number of bowel movements, stool consistency, presence of blood in stool, severity of abdominal pain, as well as in surrogate markers of disease activity following treatment. Out of 8513 initially identified articles, 22 studies involving 45,572 IBD patients met the inclusion criteria. Antidepressants, particularly bupropion, tricyclic antidepressants, selective serotonin reuptake inhibitors (SSRIs), venlafaxine, and duloxetine, were associated with improvements in IBD activity scores, including Crohn’s Disease Activity Index (CDAI) and Simple Endoscopic Score for Crohn’s Disease (SES-CD) for CD, Mayo score and Ulcerative Colitis Endoscopic Index of Severity (UCEIS) for UC. Case reports highlighted potential benefits of pregabalin and lithium carbonate, respectively, showed by the reduction in clinical and endoscopic score of disease activity for pregabalin and improvement of UC symptoms for lithium carbonate, while topiramate showed limited efficacy. Clonidine and naltrexone determined the reductions in clinical and endoscopic score of disease activity, including CDAI and Crohn’s disease endoscopy index severity score (CDEIS) for CD and Disease Activity Index (DAI) for UC. Despite the limited data and study heterogeneity, antidepressants, naltrexone, and clonidine were associated with improvements in IBD activity. Larger, prospective studies are needed to confirm the therapeutic potential of psychiatric medications in modulating IBD activity and to guide integrated clinical management.

## 1. Introduction

Symptoms of depression and anxiety, as well as perceived distress are common comorbid conditions in patients suffering from inflammatory bowel diseases (IBDs), often leading to health-related decreased quality of life and lower adherence to therapeutic programs [1,2,3]. Recent systematic reviews and meta-analyses suggested an estimated prevalence of anxiety disorders in IBD patients of 20.7%, whereas symptoms of anxiety reached 35.1% of patients [4]. Depression disorders are slightly less prevalent, with rates of 15.2% of IBD patients [4], whereas symptoms of depression affect up to 25.2% of patients [5]. Growing evidence indicate an association between stress and flares in IBD activity, with anxiety and depression being more predominant during flares [6]. IBD course and mental health seem to have a bidirectional relationship and to be reciprocally influenced by the following: patients with IBD and symptoms of depression or anxiety are at increased risk of poorer outcomes, therapy escalation, hospitalization, emergency department attendance, and increased disability whereas the presence of a more severe IBD at baseline appears to be associated with the development of symptoms of depression and anxiety [7]. Furthermore, the literature reports cases of psychiatric adverse events following treatment with the biologic drugs currently used for the treatment of IBD (e.g., infliximab), even within a few hours after the drug administration [8].

Consequently, managing psychiatric symptoms associated with gastrointestinal medical conditions, including those resulting from therapy, is becoming increasingly relevant for all healthcare professionals involved in the care of IBD patients [9].

Despite the relative paucity of supporting evidence regarding the pharmacological intervention for psychiatric symptoms, approximately 30% of IBD patients are treated with antidepressants, especially selective serotonin reuptake inhibitors (SSRIs) and tricyclic antidepressants (TCAs) [10,11]. This number was even higher in a large-scale survey conducted in Australia, where 47% of the CD patients reported current use of antidepressants and 25% believed that the treatment ameliorated their IBD [12]. Similarly, the use of anxiolytics, sedatives, and hypnotics is reported in 26% of IBD patients, a rate significantly higher than that observed in the general population [10]. Antidepressants have been shown to effectively improve anxiety, depression, and the quality of life in IBD patients. Additionally, they contribute to reducing disease activity and alleviating gastrointestinal symptoms, collectively exerting a positive impact on the disease course of IBD [13].

In this systematic review, we comprehensively collected all clinical evidence in the literature on the effectiveness of psychiatric therapies in the management of gastrointestinal symptoms of IBD patients. Our focus extended beyond addressing psychiatric comorbidities to encompass the management of IBD itself. Accordingly, the primary objective of our review was to determine the influence of these treatments on specific disease activity outcomes in IBD patients. Secondarily, we discussed the efficacy of these medications on psychiatric outcomes, while also providing practical recommendations regarding the use of specific drugs that may worsen gastrointestinal symptoms.

## 2. Material and Methods

**Figure 1:**
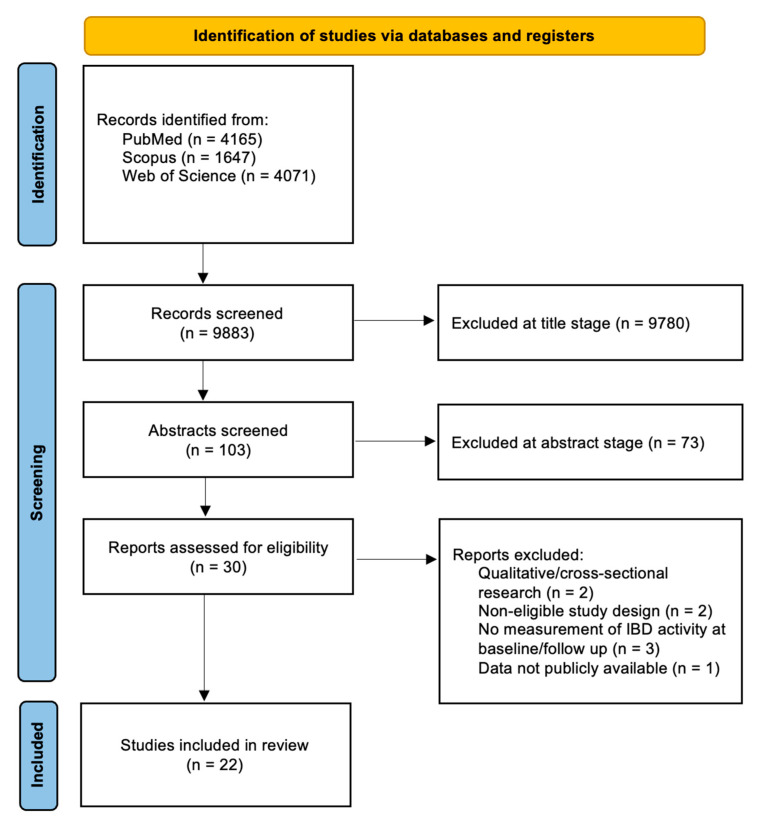
PRISMA flow diagram describing the paper selection process.

We conducted this systematic review in accordance with the Preferred Reporting Items for Systematic Reviews and Meta-Analyses (PRISMA) guidelines [14] (Figure 1).

### 2.1. Eligibility Criteria

Original peer-reviewed reports, clinical trials, retrospective and prospective cohort studies, meta-analysis, systematic reviews, and case–control studies in the English language were eligible for inclusion. The Population, Intervention, Comparison and Outcome (PICO) characteristics [15] were as follows: (1) patients diagnosed with IBD, including Crohn’s disease, ulcerative colitis, and IBD-unclassified (IBD-U); (2) use of psychiatric medications, including antidepressants, antipsychotics (typical and atypical), anticonvulsants, sedative-hypnotics, anxiolytics, stimulants, anti-craving and addiction medications, mood stabilizers, and others (e.g., Clonidine, Modafinil, Suvorexant, etc.); (3) patients receiving standard IBD treatment without psychiatric medications; and (4) changes in gastroenterological (GI) outcomes, including variations in commonly used clinical and endoscopic scores for CD and UC (e.g., Partial Mayo Score (PMS) [16], Harvey-Bradshaw index (HBI) [17], Crohn’s Disease Activity Index (CDAI) [18], Inflammatory Bowel Disease Questionnaire (IBDQ) [19], Lichtiger Colitis Activity Index (LCAI) [20,21], Endoscopic Mayo score (EMS) [16], Ulcerative Colitis Endoscopic Index of Severity (UCEIS) [22], Simple Endoscopic Score for Crohn’s Disease (SES-CD) [23], Disease Activity Index (DAI) [24], etc.), number of bowel movements, stool consistency, presence of blood in stool, severity of abdominal pain, as well as in surrogate markers of disease activity, such as IBD-related hospitalization, IBD-related surgery, or step-up medication in terms of a redeemed prescription of corticosteroids, immunomodulators or biotechnological therapies.

Clinical studies that evaluated exclusively the post-treatment variation in the quality of life, the psychological symptoms or in any psychiatric outcome of IBD patients were not included in the study. Studies evaluating patients with irritable bowel syndrome (IBS), or other GI disorders different from IBD were excluded, as well as those including patients with an uncertain diagnosis of an IBD. In the case of mixed cohorts, only data from patients that met our eligibility criteria were taken into account. We did not include animal model studies or non-original reports. Because of the likely scarcity of studies with adequate sample size, also case series with less than 10 patients and case reports were considered, without restrictions on publication year. We did not include data presented only as abstracts at conferences.

### 2.2. Information Sources and Search Strategy

A literature search was performed using the following electronic databases: Web of Science (ISI), PubMed, and SCOPUS. The last search was run on 31 July 2024. The terms “inflammatory bowel disease”, “ulcerative colitis”, “Crohn’s disease”, “IBD”, “UC”, “CD” were matched with the following drugs: Acamprosate OR Agomelatine OR Alprazolam OR Amisulpride OR Amitriptyline OR Amoxapine OR Amphetamine OR Aripiprazole OR Armodafinil OR Asenapine OR Atomoxetine OR Benztropine OR Blonanserin OR Bremelanotide OR Brexanolone OR Brexpiprazole OR Buprenorphine OR Bupropion OR Buspirone OR Caprylidene OR Carbamazepine OR Cariprazine OR Chlordiazepoxide OR Chlorpromazine OR Citalopram OR Clomipramine OR Clonazepam OR Clonidine OR Clorazepate OR Clozapine OR Cyamemazine OR Desipramine OR Desvenlafaxine OR Deutetrabenazine OR Dextromethorphan OR Diazepam OR Diphenhydramine OR Disulfiram OR Donepezil OR Dothiepin OR Doxepin OR Duloxetine OR Escitalopram OR Esketamine OR Estazolam OR Eszopiclone OR Flibanserin OR Flumazenil OR Flunitrazepam OR Fluoxetine OR Flupenthixol OR Fluphenazine OR Flurzepam OR Fluvoxamine OR Gabapentin OR Galantamine OR Fuanfacine OR Haloperidol OR Hydroxyzine OR Iloperidone OR Imipramine OR Isocarboxazid OR Ketamine OR Lamotrigine OR Lemborexant OR Levetiracetam OR Levomilnacipran OR Lisdexamfetamine OR Lithium OR Lofepramine OR Lofexidine OR Loflazepate OR Lorazepam OR Loxapine OR Lumateperone OR Lurasidone OR Maprotiline OR Memantine OR Methylfolate OR Methylphenidate OR Mianserin OR Midazolam OR Milnacipran OR Mirtazapine OR Moclobemide OR Modafinil OR Molindone OR Nalmefene OR Naltrexone OR Naltrexone/Buproprion OR Nefazodone OR Nortriptyline OR Olanzapine OR Oxazepam OR Oxcarbazepine OR Paliperidone OR Paroxetine OR Perospirone OR Perphenazine OR Phenelzine OR Phentermine/Topiramate OR Pimavanserine OR Pimozide OR Pipothiazine OR Pitolisant OR Prazosin OR Pregabalin OR Propranolol OR Protriptyline OR Quazepam OR Quetiapine OR Ramelteon OR Reboxetine OR Risperidone OR Rivastigmine OR Selegiline OR Sertindole OR Sertraline OR Sildenafil OR Sodium oxybate OR Solriamfetol OR Sulpiride OR Suvoxerant OR Tasimelteon OR Temazepam OR Thioridazine OR Thiothixene OR Tiagabine OR Tianeptine OR Topiramate OR Tranylcypromine OR Trazodone OR Triazolam OR Trifluoperazine OR Trihexyphenidyl OR Triiodothyronine OR Trimipramine OR Valbenazine OR Valproate OR Varenicline OR Venlafaxine OR Vilazodone OR Vortioxetine OR Zaleplon OR Ziprasidone OR Zolpidem OR Zonisamide OR Zopiclone OR Zotepine OR Zuclopenthixol. All terms were searched both as keywords and Medical Subject Headings (MeSH). The bibliographies of relevant (according to titles and abstracts) articles were hand-searched to provide additional references.

### 2.3. Study Selection and Data Collection Process

Three reviewers, F.D.V., E.C, and A.M.D., blinded to the other reviewer’s decision, evaluated the titles and abstracts of the studies to ascertain their eligibility. They verified separately that all the inclusion and exclusion criteria were met. In cases that were not apparent, the entire text of the publications was retrieved and examined. In every instance where there was a disagreement, a fourth author (G.C.) arbitrated.

The same three reviewers (F.D.V., E.C., and A.M.D.) separately retrieved data from qualifying studies, which were then cross-checked. Disputes were rectified by agreement. Articles that combined newly enrolled patients with those from prior studies were only taken into account for the latter. For mixed cohorts, the study only included patient data that satisfied our inclusion and exclusion criteria. Due to methodological heterogeneity and the variability in outcomes assessed across the included studies—ranging from clinical to endoscopic and including both direct and surrogate endpoints—we chose not to perform a meta-analysis or any formal statistical synthesis. Instead, we conducted a descriptive analysis, discussing each study individually in terms of design, methodology, and outcomes. For this reason, we did not register a review protocol on PROSPERO. We chose to report our results in four sections according to the type of drug that was administered.

We collected study references and citations in the EndNote software application version 20 (Thomson Reuters, New York, NY, USA). A data collection form was designed in Microsoft Excel 2016 (Microsoft, Redmond, WA, USA).

## 3. Results

A total of 8513 studies (4165 on PubMed, 4071 on Web of Science and 1647 on Scopus) were identified overall using the abovementioned research strategies. A total of 103 articles were selected for the full abstract screening, and after that 22 articles were chosen for the synthesis of results, including 7 RCTs, 4 non-randomized controlled clinical trial, 6 observational studies, 5 case series/reports. All selected studies were in English. The 22 articles covered a population of 45.572 IBD patients, including CD, UC and IBD-U. All included studies utilized different assessment tools to evaluate both gastrointestinal and psychiatric outcomes, reflecting differences in research objectives and study populations. A summary of the primary outcome measures employed is provided in Supplementary Table S1.

Supplementary Table S2 summarizes the mechanisms of action, primary indications according to the Food and Drug Administration (FDA), and major gastrointestinal side effects of the main drugs included in the manuscript. Additionally, the table provides detailed insights and further information regarding the administration of these medications.

### 3.1. Antidepressants

Among the 22 selected studies, 12 were centered on the investigation of antidepressant therapies, including 4 RCTs, 5 observational cohort studies and 3 case series/reports. Four out of 12 studies (33%) investigated different antidepressant drugs (SSRIs, TCAs, serotonin noradrenergic reuptake inhibitors (SNRIs), bupropion, trazodone and mirtazapine), while 2 (17%) focused on bupropion, 1 (8%) on tianeptine, 1 (8%) on venlafaxine, 1 (8%) on phenelzine, 1 (8%) on fluoxetine, 1 (8%) on duloxetine, and 1 (8%) on TCAs. Overall, SSRIs and SNRIs constituted the most frequently prescribed classes of antidepressants.

In details, two prospective and two retrospective observational studies investigated the role of several classes of antidepressant, including SSRI, SNRI, TCAs, and trazodone, on the disease course in patients affected by IBDs [25]. Notably, in a prospective study of 331 IBD patients, Hall et al. observed a trend toward lower rates of escalation of medical therapy among patients receiving antidepressants at baseline, and of disease flares, hospitalization and intestinal resection rates in those with abnormal anxiety or depression scores at study entry [25]. Similarly, Kristensen et al.’s retrospective population-based cohort study, encompassing 42,890 IBD patients, underscored a significantly reduced relapse rate in individuals exposed to antidepressants compared to their non-exposed counterparts, with a more pronounced effect observed in CD patients than in UC patients. Additionally, patients with no prior antidepressant usage exhibited superior disease course outcomes upon antidepressant exposure compared to non-users [26]. Yanartas et al.’s prospective cohort study, involving 67 IBD patients treated with various antidepressant classes, evidenced significant improvements in hemoglobin levels and CDAI scores compared with baseline among individuals who adhered rigorously to the prescribed 6-month treatment regimen [27]. Furthermore, a retrospective case–control study, including 58 IBD patients, revealed that patients receiving various antidepressant medications, particularly citalopram and fluoxetine, experienced fewer relapses and corticosteroid courses in the year after antidepressant initiation than in the year before; these findings were absent in the control group [28]. Conversely, in two different surveys (2012, 2014), Mikoc-Walus et al. investigated the efficacy of several antidepressants in IBD patients concurrently undergoing treatment for depression or anxiety, revealing that 67% of patients reported no alteration in their IBD activity, 26% reported physical symptom improvement, 5% referred symptom exacerbation, and 2% attributed difficulty in distinguishing antidepressant effects from other potential causal factors [29].

A randomized clinical trial (RCT) conducted by Daghaghzadeh et al. established the efficacy of duloxetine in alleviating symptom severity, as assessed by the LCAI score, in 35 individuals diagnosed with IBD [30]. Meanwhile, Mikocka-Walus et al. (2017) examined the utility of fluoxetine, an SSRI, in CD patients, revealing no significant improvement of CDAI scores or fecal calprotectin levels after the treatment [29]. Recently, Liang et al. conducted an RCT, exploring the therapeutic potential of venlafaxine, an SNRI, in 45 patients diagnosed with UC and CD. The study’s primary endpoints included the IBDQ score and disease activity assessments (CDAI for CD, Mayo score for UC), with secondary endpoints encompassing disease course, SES-CD, UCEIS, relapse rate, corticosteroid/biologic usage frequency, as well as pertinent laboratory parameters, including white blood cells count (WBC), albumin, C reactive protein (CRP), fecal calprotectin, erythrocyte sedimentation (ESR), tumor necrosis factor (TNF)-α, and interleukin (IL)-10. Notably, IBDQ scores exhibited a significant elevation in the venlafaxine cohort compared to the placebo group. Furthermore, UC and CD patients treated with venlafaxine demonstrated reduced Mayo score and CDAI score, respectively, following the 6-month assessment period. However, the study did not discern significant disparities in UCEIS and SES-CD scores between the venlafaxine and placebo cohorts at the 6-month juncture [31].

Only one retrospective cohort investigation focused on the efficacy of tricyclic antidepressants (TCAs) in ameliorating gastrointestinal symptoms in IBD patients, elucidating a noteworthy improvement in the severity scores (established Likert scales) of both UC and CD cohorts. However, UC patients presented a better response to TCA that those with CD [32].

Tianeptine, an atypical antidepressant, succeeded in reducing symptoms of anxiety and depression, as well as disease activity index, in 60 patients with IBD compared with placebo after 12 months [33].

Interestingly, some case reports exhibited promising effects on gastrointestinal symptoms after bupropion intake. A case series of 4 patients by Kane et al. (2003) and a case report of 2 patients by Kast and Altschuler (2001) documented the achievement of clinical remission (CDAI < 150) in six patients affected by CD following bupropion administration for varied indications [34,35]. One patient experienced a flare-up of CD after discontinuing bupropion on her own.

Finally, in 1998, Kast et al. detailed the case of a 33-year-old woman receiving phenelzine, who experienced sustained improvement in bowel function and abdominal pain, persisting post-azathioprine and prednisone tapering, albeit experiencing CD relapse subsequent to nortriptyline transition six weeks later [36].

Only a minority of the aforementioned studies specifically examined also the impact of antidepressants on psychiatric disorders in patients with IBD. A population-based study conducted by Jayasooriya (2022) revealed a higher incidence of antidepressant usage within the IBD cohort, with the greatest risk occurring during the initial year post-IBD diagnosis [37]. Furthermore, among IBD patients initiating antidepressant therapy, 67% received treatment for a duration less than the recommended minimum of 7 months.

The RCT featuring duloxetine by Daghaghzadeh et al., evaluated anxiety and depression with the Hospital Anxiety and Depression Scale (HADS), while Quality of Life (QOL) was assessed via the World Health Organization Quality of Life abbreviated version (WHOQOL-BREF) [30]. The findings indicated a significant reduction in anxiety and depression levels alongside an enhancement in physical, psychological, and social QOL domains, although no notable difference in environmental QOL was observed. Similarly, the randomized clinical trial conducted by Mikocka-Walus et al. (2017), involving fluoxetine, utilized the same assessment scales, yet fluoxetine exhibited negligible influence on psychological symptoms or QOL [29].

HADS was further employed in an additional RCT featuring an SNRI, venlafaxine (Liang, 2022), which revealed a noteworthy reduction in HADS depression scores after the treatment [31]. Additionally, a prospective cohort study [27] (Yanartas, 2016), predominantly comprising SSRI recipients, reported improvements in anxiety and depression levels as measured by HAD-A and HAD-D scales, respectively, alongside enhancements in SF-36 and ASSES scores.

In two case reports by Kast and Altschuler (2001) [35] and Kast (1998) [36], bupropion and phenelzine exhibited efficacy in alleviating depression symptoms in patients with CD.

Data extracted from each primary study are shown in Table 1.

### 3.2. Antiepileptics

Only one retrospective cohort study [38] focused on topiramate use in IBD patients, based on a large cohort of IBD patients: 775 were treated with topiramate and 958 were treated with other anticonvulsant and anti-migraine medications. However, there was no difference between topiramate users concerning steroid prescription, post-exposure initiation of biologic agents, abdominal surgery or hospitalization. In this study, effects on psychological symptoms were not reported.

Interestingly, one case report described a clinical, biochemical, and subsequently endoscopic (after 6 months) improvement in a patient with CD undergoing therapy with Pregabalin for psychic and physical anxiety [39].

Data extracted from each primary study are shown in Table 2.

### 3.3. Hypnoinducers and Anxiolytics

According to a retrospective matched cohort study [40] (Bernstein, 2022), the use of BZD and Z-drugs (to treat mood/anxiety disorders and sleep disorders) is more common in IBD patients than in controls. Nevertheless, all the available studies focus exclusively on psychological effects, not including gastrointestinal outcomes (Stokes, 1978) [41], therefore none of them met our eligibility criteria.

### 3.4. Mood Stabilizers

Only one case report met our eligibility criteria, [42] describing the administration of lithium carbonate in the manic phase of a 67-year-old patient with ulcerative colitis, in an active phase with recent endoscopic evidence of “severe, active ulcerative colitis, already undergoing therapy with salicosulfapiridine (azulfadine)”. After five days, there was considerable improvement in both gastrointestinal and psychiatric symptoms, and after two months, the endoscopic evidence revealed considerable improvement with only a few small bleeding spots. Data extracted from the primary study are shown in Table 3.

### 3.5. Others

Other studies which met our eligibility criteria investigated the role of naltrexone, clonidine and lithium on the disease course of IBD patients. A total of four trials explored the effect of naltrexone in IBD, three of them focused on CD.

Lie et al. 2018 [43] evaluated low-dose naltrexone (LDN) for induction of remission in IBD patients (UC and CD) not responsive to conventional therapy. LDN induced clinical improvement in 74.5% and remission in 25.5% of patients. There was no statistically significant difference between CD and UC patients in rates of response or remission. Patients achieving clinical remission had a significantly greater improvement in endoscopic score than patients not reaching clinical response. However, 14.9% reported adverse events due to LDN (vivid dreams, drowsiness, and headache).

Smith et al. in 2011 [44] in an RCT, confirmed naltrexone as a promising therapy for CD: 88% of those treated with naltrexone had at least a 70-point decline in CDAI scores compared to 40% of placebo-treated patients, and 78% of active arm had an endoscopic response (a 5-point decline in the Crohn’s disease endoscopy index severity score (CDEIS) from baseline compared to 28% response in placebo-treated controls, thereby confirming their findings previously described in 2007. In addition, in 2007, Smith et al. [45] also reported an improvement in QoL compared to baseline during naltrexone therapy. Smith et al. randomized pediatric patients with CD [46] to placebo or LDN in another study. Naltrexone was well tolerated without any serious adverse events, and the PCDAI (Pediatric Crohn’s Disease Activity Index) significantly decreased in the active arm, with 25% of those achieving remission. According to the Impact III survey, an improvement in QoL from baseline was reported.

Regarding clonidine, two studies have investigated the efficacy of clonidine in patients with UC based on drug-mediated reduction of overall enhancement of sympathetic disease activity. Lechin et al. [47] conducted a double-blind clinical trial of UC patients with severe activity, which was randomized to treatment with prednisone, sulfasalazine, or clonidine. Comparing performance among the three groups, treatment with prednisone and treatment with clonidine were significantly more effective than sulfasalazine (*p* < 0.001) for both. There were no significant differences between prednisone treatment and clonidine treatment. However, clonidine treatment induced more rapid therapeutic effects than prednisone, without significant adverse effects. Later, Furlan et al. [48] tested the reduction in sympathetic activity by clonidine on clinical changes in UC. In addition to autonomic assessment (more information in table), they were randomly assigned to transdermal clonidine for eight weeks or placebo. Changes in the autonomic profile after clonidine were associated with reduced DAI score (concomitant amelioration of symptoms and endoscopic pattern). Normalization of the autonomic profile with clonidine was accompanied by improved disease and a general increase in sympathetic activity characterized by active UC.

Data extracted from each primary study are shown in Table 4.

## 4. Discussion

**Figure 2:**
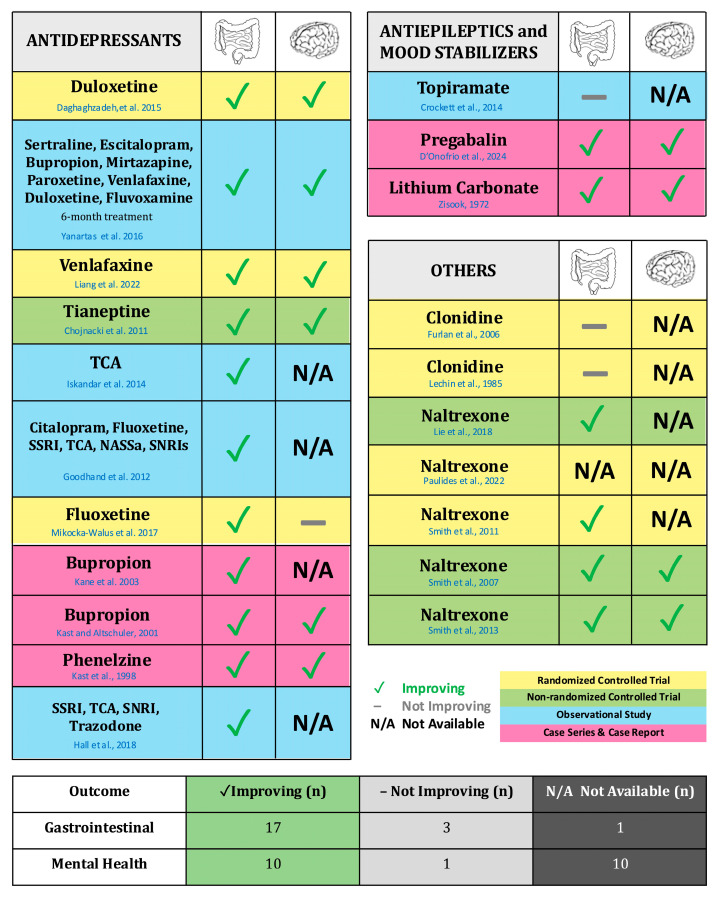
Graphical summary of the studies included in the systematic review. This table summarizes the key studies included in this review, highlighting the effectiveness of certain psychotropic drugs on both gastrointestinal and psychiatric outcomes. The studies are organized by drug class—Antidepressants (Daghdhaghzadeh et al., 2015 [30], Yanartaş et al., 2016 [27], Liang et al., 2022 [31], Chojnacki et al., 2011 [33], Iskandar et al., 2014 [32], Goodhand et al., 2012 [28], Mikocka-Walus et al., 2017 [29], Kane et al., 2003 [34], Kast and Altschuler, 2001 [35], Kast et al., 1998 [36], Hall et al., 2018 [25]), Antiepileptics and Mood Stabilizers (Crockett et al., 2014 [38], D’Onofrio et al., 2024 [39], Zissok, 1972 [42]), and Others (Furlan et al., 2006 [48], Lechin et al., 1985 [47], Lie et al., 2018 [43], Paulides et al., 2022 [49], Smith et al., 2011 [44], Smith et al., 2007 [45], Smith et al., 2013 [46]). The study by Kristensen et al., 2019 [26] was not included because there are no psychiatric/gastroenterological outcomes available for specific psychotropic drugs, but only for classes of drugs (antidepressants). Note: CR (Case-report); CS (Case-series); NASSa (Noradrenaline and specific serotonergic antidepressants); SNRI (Serotonin and norepinephrine reuptake inhibitors); SSRI (Selective serotonin reuptake inhibitors); TCA (Tricyclic antidepressants).

In Western countries, between 10 and 30% of patients with IBD take antidepressant medications; nonetheless, to date only a few studies have investigated their role in the management of IBDs [50,51,52]. Various systematic and narrative reviews have consistently acknowledged a favorable impact of antidepressants on the general well-being of patients with IBD [11,13,53,54,55]. However, these reviews underscore the constrained and low-quality evidence available, leading to the conclusion that the efficacy of antidepressants in modulating IBD activity remains indeterminate. Although our research identified very few clinical trials that could establish a causal relationship between certain medications and the physical and mental health of patients with IBD, our systematic review revealed that antidepressant use might be beneficial to the disease course in patients with CD and UC, particularly for those who did not use antidepressants before the IBD onset, or with latent subclinical symptoms of anxiety and depression [26] (Figure 2).

To date, only four placebo-controlled trials assessing the efficacy of antidepressants in IBD have been conducted, with one of these trials being nonrandomized [29,30,33]. Tianeptine, an atypical antidepressant, demonstrated a reduction in symptoms of anxiety and depression, as well as a decrease in disease activity index [33]. Duloxetine exhibited a reduction in anxiety and depression scores, an improvement in quality of life, and a decrease in clinical disease activity indices in a cohort of IBD patients [30]. Likewise, venlafaxine was shown to enhance quality of life, decrease depression scores, and reduce clinical disease activity indices (CDAI and Mayo scores) [31]. Conversely, fluoxetine did not demonstrate any significant effect on anxiety, depression, quality of life, or clinical disease activity indices. Nonetheless, modest effects of fluoxetine on immune functions have been observed, including an increased proportion of effector memory T helper cells and a decreased proportion of effector memory T cytotoxic cells [29]. Notably, antidepressant use in patients with IBDs has been associated with several positive outcomes according to observational studies and case reports, including lower rates of hospitalization, surgical intervention, and medical treatment escalation [26]. Smaller studies corroborated these findings [34,37], showing reduced medical therapy escalation and fewer disease relapses among patients receiving antidepressants [28], particularly in those with high baseline anxiety or depression [25]. Tricyclic antidepressants specifically show a better response in ulcerative colitis compared to Crohn’s disease, and bupropion has demonstrated efficacy in inducing clinical remission in patients with Crohn’s disease [32]. Furthermore, in a large epidemiological investigation encompassing over 400,000 individuals, the heightened risk of new-onset IBD observed among individuals with baseline depression appeared to be attenuated by the administration of antidepressants [56].

**Figure 3:**
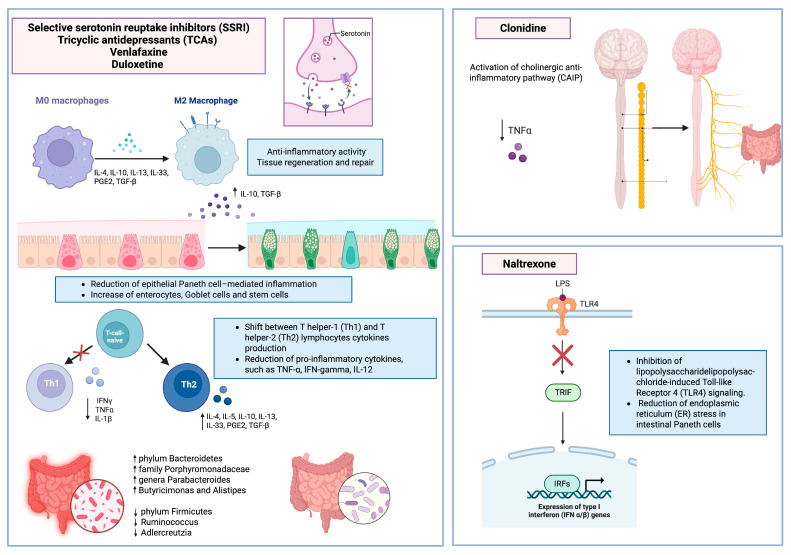
Molecular and immunological mechanisms underlying the anti-inflammatory effects of selected psychiatric medications in inflammatory bowel disease. Selective serotonin reuptake inhibitors (SSRIs), tricyclic antidepressants (TCAs), venlafaxine, and duloxetine contribute to inflammation modulation through the promotion of M2 macrophage polarization, increased production of anti-inflammatory cytokines (e.g., IL-10, TGF-β), reduction in Paneth cell-mediated epithelial inflammation, and modulation of gut microbiota composition. These drugs also induce a Th1-to-Th2 cytokine shift, reducing pro-inflammatory mediators such as TNF-α, IL-1β, and IFN-γ. Naltrexone inhibits lipopolysaccharide (LPS)-induced TLR4 signaling, reducing ER stress in Paneth cells and the expression of pro-inflammatory interferon genes. Clonidine activates the cholinergic anti-inflammatory pathway (CAIP), resulting in decreased TNF-α levels via autonomic nervous system modulation.

It is well established that the vagus nerve, which is a key component of the brain–gut axis, plays a critical role in the tonic suppression of acute inflammation in IBD. Depression has been shown to disrupt this tonic vagal inhibition of pro-inflammatory cells, thereby increasing vulnerability to intestinal inflammation. Tricyclic antidepressants have been demonstrated to alleviate intestinal inflammation by restoring vagal function [57]. Additional studies have reported that depression can reactivate dormant chronic colitis through a process dependent on the α7 subunit of the nicotinic acetylcholine receptor (α7nAChR), which is normalized following treatment with antidepressants [58]. Indeed, the most intriguing potential effect of antidepressants in IBD is for the control of intestinal and systemic inflammation. A recent meta-analysis [59] showed that an antidepressant treatment lowers the levels of the pro-inflammatory cytokines IL-4 and IL-6 in peripheral blood, thus resulting in an anti-inflammatory effect. The immunomodulatory mechanism of antidepressants could also be based on a shift between T helper-1 (Th1) and T helper-2 (Th2) lymphocytes cytokines production [60] (Figure 3). In animal models of IBD, desipramine demonstrated a reduction in microscopic damage and mitigated colonic myeloperoxidase activity compared to placebo [61]. Likewise, both desipramine and fluoxetine significantly decreased serum concentrations of IL-1β and TNF-α in an animal model (all *p* < 0.001) [62]. A recent study by Teng et al. showed that treatment with fluoxetine in C56BL/6 mice of the IBD model induced macrophages to M2-like phenotype and inhibited the production of Paneth cells, while enterocytes, goblet cells and stem cells became the dominating cells, thereby exerting a protective effect on IBD [63]. As is widely known, the main target of fluoxetine is the 5-HT transporter (SERT), which blocks reuptake and prolongs neurotransmitter signaling of 5-HT [64]. Previous data suggest that SERT inhibition may exert a systemic immunosuppressive effect, partly through stimulation of M2 macrophage polarization. Another SSRI, sertraline, has been shown to inhibit the activation of Toll-like receptor (TLR)-3, thereby interfering with innate immunity [65]. Moreover, SSRIs seem to block the activation and proliferation of antigen presenting cells, thus influencing adaptive immunity [66]. Similarly, preclinical in vivo studies suggested a potential immunosuppressive effect for SNRIs, reducing the generation of pro-inflammatory cytokines, such as TNF-α, IFN-gamma, and IL-12 [67]. However, in studies on humans and healthy volunteers, venlafaxine and duloxetine presented inconsistent and minor effects on cytokines, lymphocyte subpopulations or circulating levels of neurotrophic factors [68,69,70]. These findings collectively suggest that the use of antidepressants should be considered in patients with IBD, not only for managing subclinical or mild symptoms of anxiety and depression—often underestimated and underdiagnosed—but also for potentially modulating the disease course and improving clinical outcomes in selected patients. Although studies investigating the kynurenine pathway of tryptophan degradation in IBD are lacking, the previous literature suggested that depression linked with chronic inflammatory disorders, such as cancer, might have a distinct etiology compared to other forms of depression [71,72]. Notably, alterations in the kynurenine pathway due to inflammation, interfering with the bioavailability of tryptophan, necessary for the synthesis of serotonin, may play a crucial role in the development and persistence of both conditions [71,72].

Hence, targeting enzymes implicated in the kynurenine pathway could present a dual therapeutic opportunity for treating both IBD (or other inflammatory conditions) and depression concurrently [73]. In this scenario, antidepressants may potentially facilitate this approach [74].

Additionally, antidepressants may exert their anti-inflammatory effects through the modulation of intestinal microbiota composition [75]. Fluoxetine increases the abundance of certain taxa such as phylum Bacteroidetes, family Porphyromonadaceae, and genera Parabacteroides, Butyricimonas, and Alistipes. It decreases the abundance of phylum Firmicutes, as well as taxa like Ruminococcus and Adlercreutzia [76,77]. Escitalopram reduces the abundance of Ruminococcus and Adlercreutzia [76]. Venlafaxine and duloxetine also decrease the abundance of Ruminococcus and Adlercreutzia [76]. Amitriptyline, on the other hand, boosts the abundance of phylum Bacteroidetes, family Porphyromonadaceae, family Bacteroidaceae, and genera Parabacteroides, Butyricimonas, and Alistipes, while reducing the abundance of phylum Firmicutes [77]. Ketamine increases the abundance of bacterial genera such as Lactobacillus, Turicibacter, and Sarcina, while decreasing the abundance of opportunistic pathogens like Ruminococcus and Mucispirallum [78].

Another important role of antidepressants is in the treatment of chronic pain. In a recent meta-analysis [79], duloxetine was found to be the most effective antidepressant for managing chronic pain. There are also studies supporting the efficacy of milnacipran in treating chronic pain, but these studies are significantly fewer and of lower quality compared to those on duloxetine. The mechanism involves noradrenaline reuptake inhibition, which enhances analgesic effects mainly through α2-adrenergic receptors in the dorsal horn of the spinal cord [80]. Therefore, duloxetine might be a viable candidate for conditions involving chronic pain (such as IBD) and low mood.

A recent and intriguing meta-analysis demonstrated that antidepressants are safe and effective in treating depression comorbid with medical conditions. Additionally, the meta-analysis highlighted the ability of antidepressants to prevent depression [81]. Given the high prevalence of depression among individuals with IBD, it might be beneficial to consider low-dose antidepressants for those with a history of depressive episodes or even mild alterations on baseline psychometric scales for depression. Ultimately, the decision should depend on the clinical judgment of the physician, preferably with a psychiatrist’s consultation.

Despite various gastrointestinal side effects reported in the literature due to antidepressant therapy, particularly with escitalopram and sertraline, which seem to be less tolerated by the gastrointestinal tract [82], researchers are increasingly interested in understanding the role of antidepressants, specifically serotonin reuptake inhibitors, in functional gastrointestinal disorders like irritable bowel syndrome (IBS). Considering that IBD can cause issues with intestinal motility leading to constipation and bloating, and to the overlap between IBD and IBS [83], antidepressants may prove beneficial in managing these conditions. Notably, in a randomized controlled trial by Tack et al., it is reported that citalopram can increase colonic contractility and the occurrence of high-amplitude propagated contractions, together with an increased colonic compliance during fasting, suppressing the postprandial colonic tone [84].

Additionally, antidepressants (along with other pharmacological classes) can be used in managing psychiatric side effects associated with corticosteroid therapy [85], which is widely used in the management of IBD [86]. Symptoms seem to vary according to the corticosteroid dosage and typically emerge within the initial weeks of treatment. Unfortunately, there is a lack of knowledge regarding the risk factors associated with the onset of mood instability [87].

Another promising molecule that has been investigated in patients with IBD is naltrexone. The rationale for its use as a treatment in IBD is based on the relevance of the endogenous opioid system in gut immunity [88,89]. Indeed, in IBD patients, the μ-opioid receptor (MOR) is overexpressed in mucosal T-lymphocytes and monocytes, and the administration of low dose Naltrexone (LDN) would appear to upregulate the endogenous encephalin and endorphin levels and to have a positive modulatory effect on the MOR [90].

Additionally, it has been shown that the opioid inactive (+)-isomers of naltrexone inhibit lipopolysaccharide-induced Toll-like Receptor 4 (TLR4) signaling, a bacterial-induced inflammatory pathway contributing to IBD [91,92].

Furthermore, the effects of such therapy could also be expressed on the reduction in endoplasmic reticulum (ER) stress in intestinal Paneth cells, which is one of the contributing factors in IBD [93,94,95].

In the studies analyzed, naltrexone therapy was associated with favorable clinical response rates, clinical remission, and endoscopic response (endoscopic scores improved by 48% compared to baseline, *p* = 0.018) [44]. Naltrexone also showed good tolerance in the pediatric population with CD. However, these results were obtained from cohorts with a relatively small number of participants over a limited observation period. Although most of the studies are conducted on CD, it is not entirely clear whether this therapy may also be helpful in UC patients (only one study is available) or whether there are any subclasses of patients in which it may be more effective. A multicenter study by Paulides et al. with a more significant number of patients for a longer observation time is still ongoing [43].

In the literature, there are no studies evaluating how naltrexone can induce changes in the gut microbiota. However, there is evidence showing that naltrexone can improve inflammatory parameters in a murine model of enteritis [96].

From a psychiatric perspective, naltrexone can also enhance positive emotional states by boosting the effects of endogenous opioids, leading to increased positive feelings and energy [97]. A review by Brown and Panksepp suggests that low doses of naltrexone may also play a role in promoting stress resilience, exercise, social bonding, and emotional well-being, as well as improving psychiatric conditions such as autism and depression [98].

Clonidine, by activating the alpha-2 adrenergic receptors in the brainstem, inhibits the release of norepinephrine, a neurotransmitter that typically stimulates the sympathetic nervous system. The rationale for its utilization is based on the role of the cholinergic anti-inflammatory pathway (CAIP) on the potential anti-inflammatory effect on immune cells in animal models [99]. Experimental activation of CAIP inhibits TNF synthesis in the liver, spleen and heart, attenuating serum TNF concentrations during endotoxemia by LPS [100]. Furthermore, an overall increased sympathetic activity would seem to support the inflammatory process of UC, which can also be inferred from the fact that psychological stress can reactivate quiescent UC. On the other hand, increasing para-sympathetic organ drive using a nicotine patch has been shown to improve the inflammatory state in patients with UC [101].

However, as previously described, studies investigating clonidine administration in patients with ulcerative colitis are limited. Lechin et al. in 1985 observed a clinical and endoscopic improvement with clonidine treatment, and it was significantly more effective when compared to sulfasalazine, while there were no significant differences between prednisone and clonidine treatment [47].

Later, although they stated that their investigation must not be considered a pharmacological trial, Furlan and collaborators found that clonidine reduced systemic neural sympathetic activity and increased vagal cardiac modulation, which was associated with decreased DAI, indicated by amelioration of clinical symptoms and colon endoscopic pattern. However, several crucial variables, including smoking status, previous UC therapy, and gender, were documented but not individually examined, likely due to the limited size of the sample. The researchers did not specify whether a post hoc test was conducted during the statistical analysis. Even in this case, the number of patients treated, and the observation time are limited, so future more extensive studies are needed to aim for such an alternative or combined therapy for future clinical practice in IBD [48]. Notably, no significant hypotensive effects were reported during treatment in these studies.

Conversely, for drugs such as pregabalin and lithium carbonate, the precise mechanisms by which they may influence intestinal inflammation in IBD remain largely unclear. However, some preclinical data provide mechanistic insights. In a murine model of acetic acid-induced colitis, pregabalin reduced both macroscopic and microscopic intestinal damage and inflammation, decreasing colonic levels of TNF-α, IL-6, IL-1β, and myeloperoxidase (MPO) activity [DOI: 10.4103/1735-5362.329924]. Pregabalin appears to exert its effects via modulation of the glutamate/N-methyl-D-aspartate receptor (NMDA)–nuclear factor kappa B (NF-κB)–cyclooxygenase-2 (COX-2) signaling axis, leading to reduced cytokine secretion and neutrophil infiltration. Additionally, pregabalin may attenuate neurogenic inflammation through interaction with the alpha-2-delta (α2δ) subunits of voltage-gated calcium channels. In sensitized animal models, it has been shown to reduce accelerated defecation and increase the colonic nociceptive threshold, indicating a role in visceral pain modulation [102].

Regarding lithium, it has been shown to inhibit glycogen synthase kinase-3 beta (GSK-3β), activate the wingless-related integration site/beta-catenin (Wnt/β-catenin) signaling pathway. This activity promotes epithelial regeneration and downregulates key inflammatory mediators such as COX-2, TNF-α, and IL-1β.

Accordingly, in dextran sulfate sodium (DSS)-induced colitis models, lithium carbonate also promoted the activation of regulatory T lymphocytes (Tregs) via the G-protein-coupled receptor 43 (GPR43), contributing to the attenuation of intestinal inflammation [103]. In summary, the beneficial effects observed with antidepressants, clonidine, and naltrexone likely reflect their shared ability to modulate both central and peripheral inflammatory pathways. Antidepressants exert anti-inflammatory actions via cytokine suppression, vagal tone enhancement, and microbiota modulation. Naltrexone acts through μ-opioid receptor blockade and TLR4 inhibition, while clonidine reduces sympathetic activity and activates the cholinergic anti-inflammatory pathway. In contrast, agents like topiramate lack such immunomodulatory properties, and pregabalin or lithium although promising are supported only by limited preclinical evidence.

## 5. Strengths and Limitations

A key strength of our systematic review lies in its global perspective, achieved through an extensive and comprehensive literature search that included studies conducted across multiple countries and over an extended time frame. Notably, this is the first systematic effort to compile all available evidence, including case series and case reports, on the efficacy of psychiatric medications in managing gastrointestinal symptoms, as well as both direct and surrogate markers of disease activity in IBD.

However, our review has certain limitations that should be acknowledged. The studies included encompass a wide range of designs—randomized and nonrandomized controlled trials, observational studies (both prospective and retrospective), case series, and case reports—which exhibit significant heterogeneity in study design, study population, intervention, definition of outcome and in the modality of outcome measurement. This heterogeneity precluded the possibility of performing a meta-analysis, limited the statistical power and impaired the overall level of evidence derived from this review.

## 6. Conclusions

This systematic review demonstrates the potential of several classes of psychiatric medications to improve both gastroenterological outcomes, such as inflammation and disease activity, and psychological well-being in IBD. Antidepressants, including bupropion, TCAs, SSRIs, venlafaxine, and duloxetine, have been consistently associated with improvements in both direct and surrogate markers of disease activity. While case reports suggest potential benefits from pregabalin and lithium carbonate, no improvement in disease activity has been observed with topiramate. Moreover, some RCTs have reported positive outcomes with clonidine and naltrexone in both UC and CD.

Our review also highlights the mechanisms of these psychotropic drugs that extend beyond the “simple” neurochemical modulation within the central nervous system, also contributing to an anti-inflammatory action, which may in the future allow their use as adjuvant anti-inflammatory therapies. These findings suggest that a holistic approach, integrating psychiatry with other medical disciplines in IBD care, holds promise for improving disease activity, clinical and surgical outcomes, and quality of life in IBD patients. However, further robust RCTs with larger sample sizes and standardized outcome measures, along with translational research, are necessary to evaluate whether psychiatric medications with well-established safety profiles could serve as adjuvant treatments in IBD management or function as anti-inflammatory and immunomodulatory agents.

## Figures and Tables

**Table 1:** Included studies on antidepressants for gastrointestinal outcomes in IBD patients.

				Measurement and Assessment		Results		
Author (Year), Ref.	Drug	Study Population	Study Design	IBD	Psychiatric Condition	IBD	Psychiatric Condition	Follow-Up
Daghaghzadeh, et al. (2015) [30]	Duloxetina	35 adult IBD patients (no flare up in the previous 6 months) CD = 13 UC = 22	Placebo-controlled RCT: Study group (n = 17) treated with duloxetine 30–60 mg daily for 12 weeks. Control group (n = 18) treated with placebo. Both groups also received a stable dose of mesalazine	The severity of symptoms was assessed with LCAI	Anxiety and depression were assessed with HADS. The QOL was assessed with WHOQOL-BREF.	Severity of symptoms was significantly decreased in the duloxetine group compared to the placebo group.	Depression and anxiety were significantly decreased in the duloxetin group compared to the placebo group. Physical, psychological, and social QoL were significantly increased after treatment with duloxetine.	12 weeks
Yanartas et al. (2016) [27]	Sertraline 21.0%, escitalopram 15.8%, bupropion 12.3%, mirtazapine 12.3%, paroxetine 10.6%, venlafaxine 5.2%, duloxetine 3.5%, fluvoxamine 1.8%, antidepressant combination treatment	67 patients with IBD and anxiety and/or a depression disorder CD = 31 UC = 36	Prospective cohort study: Patients were divided into 2 groups: Group A (47 patients who had completely adhered to the 6-month drug treatment Group B (20 patients who were nonadherent)	CDAI and modified MMS were used for the assessment of disease activity in patients with CD or UC, respectively, along with CRP, complete blood count, and routine blood biochemistry	Anxiety and depression were assessed with HADS. SF-36 and ASEX tests were used to assess QoL and sexual dysfunction	Hemoglobin plus CDAI parameters were statistically improved at the final visit in group A	HAD-Anxiety (HAD-A), HAD-Depression (HAD-D), all domains of SF-36, ASEX were statistically improved at the final visit in group A.	6 months
Iskandar et al. (2014) [32]	TCA	81 patients with IBD in clinical remission/mild activity and IBS UC = 23 CD = 58	Retrospective study: IBD patients in clinical remission/with mild inflammation but with persistent GI symptoms were treated with TCA (10–150 mg).	Established Likert scales were used to score baseline symptom severity (0 = no symptoms, 3 = severe symptoms) and TCA response (0 = no improvement; 3 = complete satisfaction).	N/A	Baseline severity scores (CD: 2.07 ± 0.03, UC: 2.03 ± 0.04, *p* = 0.67), UC patients responded significantly better to TCA therapy, with a Likert response score of 1.86 ± 0.13 for UC and 1.26 ± 0.11 for CD (*p* = 0.003). 83% of UC patients had at least a moderate symptomatic improvement on TCA, compared with 50% of CD patients (*p* = 0.01).	N/A	11 years
Goodhand et al. (2012) [28]	Citalopram 20 mg (20–60 mg) and fluoxetine 20 mg (20–60 mg) were the most used. Others were SSRIs, TCA, NaSSa, and SNRIs.	58 IBD patients divided into 2 groups (n = 29 IBD patients on antidepressant, and n = 29 IBD control patients not on antidepressant). Patients were treated also with corticosteroids, 5-ASA, immunosuppressive agents and anti-TNF-α. UC = 14 CD = 15 (in each group)	Retrospective case–control study: Comparison of the course of IBD patients during the year before (year 1) and after (year 2) they were started on an antidepressant to that of controls matched for age, sex, disease type, medication at baseline, and relapse rate in year 1.	The outcome measures were the number of relapses, number of endoscopic procedures, number of hospital admissions and outpatient attendances, numbers of courses of steroids and relapse-related use of other IBD medications. Disease activity was defined as the presence of diarrhea and rectal bleeding in UC, the presence of abdominal pain and/or diarrhea and/or a CRP >10 mg/L in CD, a relapse was defined as the presence of these symptoms with a step-up in IBD medication.	N/A	Patients had fewer relapses and courses of steroids in the year after starting an antidepressant than in the year before (1 [0–4] (median [range]) vs. 0 [0–4], *p* = 0.002; 1 [0–3] vs. 0 [0–4], *p* < 0.001, respectively); the controls showed no changes between years 1 and 2 in relapses or courses of steroids	N/A	2 years
Mikocka-Walus et al. (2017) [29]	Fluoxetina 20 mg daily	26 adult patients with clinically established diagnosis of CD, in clinical remission but who flared CD in the last 12 month.	Pilot Randomized Placebo-Controlled Trial: Study group: 14 patients treated with fluoxetine Placebo group: 12 patients. Patients in both treatment arms remained on their current IBD medication. Participants provided blood and stool samples and complete questionnaires on four occasions [baseline, 3, 6 and 12 months]	The primary outcome measures were a significant group difference in the CD remission rate as measured on the CDAI [cut-off < 150]. The secondary measure was difference on remission rates as measured by fecal calprotectin;	The primary outcome was the difference in means on the WHOQo. Secondary measures were differences between HADS.	There was no statistically significant difference in the proportion of participants in remission at any time point, using either the CDAI [F(3, 27.5) = 0.064, *p* = 0.978] or fecal calprotectin [F (3, 32.5) = 1.08, *p* = 0.371],	There was no significant group difference in physical QoL, psychological QoL, social relationships QoL or environmental QoL over the 12 months. There was no significant group difference in anxiety or depression over the 12 months.	12 months
Kane et al. (2003) [34]	Buproprion (100 mg)	4 CD patients	Case series: Bupropion (100 mg) was prescribed for depression or smoking cessation	Outcome measurements: CDAI.	Two patients were diagnosed with depression	CDAI decreased to 150 within 6 weeks without a change in other Crohn’s medications	N/A	6 weeks
Kast and Altschuler et al. (2001) [35]	Bupropion	44-year-old woman with CD, taking fluoxetine 40 mg every day for depression, and mesalamine 500 mg twice a day. 45-year-old-man with CD, taking azathioprine and fluoxetine to help with pain control (then switched to bupropion)	Case series: Female: Bupropion 150 mg 3 times daily for depression and dysthymia. Fluoxetine was stopped and mesalamine was tapered off. Male: Bupropion 150 mg 3 times daily for smoking cessation	Outcome measurements: CDAI Woman: CDAI was 202. Man: CDAI was 275.	Woman had an episode of major depression, superimposed on a chronic mild depressed state (dysthymia).	Female: 19-month remission, no CD flares since starting bupropion. She stopped buproprion on her own but had a relapse and was forced to restart buproprion. CDAI = 0. Male: CDAI = 45.	Female: major depression remitted; dysthymia remained.	Woman: 19 months Man: not reported
Kast et al. (1998) [36]	Phenelzine	33-year-old woman with CD.	Case report: Phenelzine 15 mg three times daily for one month, then 30 mg 3 times daily for 2 years.	Outcome measurements: clinical symptoms, such as diarrhea and abdominal pain.	She had an anxiety-prominent major depressive episode.	Seven days after phenelzine intake, bowel movements dropped from 10 to 3 or 4 daily without cramping. One month later, after increasing phenelzine 30 mg three times daily, she had 1 well-formed bowel movement daily and no cramping. Azathioprine and prednisone were tapered off. Two years after she switched to nortriptyline and after 6 weeks was admitted to the hospital with CD relapse.	Depression responded well.	2 years
Liang et al. (2022) [31]	Venlafaxine	45 patients with IBD were included UC = 21 CD = 25	Prospective, randomized, double-blind, and placebo-controlled clinical trial. IBD patients with symptoms of anxiety or depression were randomly assigned to receive either venlafaxine 150 mg daily or equivalent placebo and followed for 6 months (25 received venlafaxine and 20 placebo). IBDQ, Mayo score, CDAI, HADS and blood examination were completed before the enrollment, during, and after the follow-up.	The primary outcome measures were as follows: the IBDQ score and CDAI for CD and Mayo score for UC at the onset, 3 months, and 6 months, which were analyzed in the complete case population. The secondary outcome measures were the disease course, SES-CD, UCEIS, relapse rate, frequency of corticosteroids/biologics use, and laboratory parameters (WBC, ALB, CRP, ESR, TNF-α, IL-10) between the venlafaxine and placebo groups	Psychiatric outcome measures were the means on the Hospital Anxiety Depression Scale (HADS, measured on three occasions.	IBDQ scores were significantly higher in the venlafaxine group compared with placebo group (3 months: comparison of effect size:0.59, *p* = 0.005; 6 months: comparison of effect size: 1.19, *p* < 0.001. UC patients with venlafaxine had lower Mayo score than that in placebo group after the 6-month assessment (comparison of effect size: −1.47, *p* < 0.001). Venlafaxine showed significant decrease in CDAI scores compared with placebo at 6 months (comparison of effect size: −0.87, *p* = 0.006). No significant differences were observed between the 2 groups in 3 months. UCEIS (1.9 vs. 1.7, *p* = 0.249) and SES-CD (4.6 vs. 8.6, *p* = 0.071) showed no significant differences between the venlafaxine group and placebo group at 6 months.	A significant reduction in the HADS depression scores was observed between the two groups both at 3 months (6.41 vs. 8.81, *p* < 0.001) and at 6 months	6 months
Kristensen et al. (2019) [26]	Antidepressants (SNRI, SSRI, TCA, Mirtazapine and others)	42,890 IBD patients were included (UC: 69.5%; CD: 30.5%).	Population-based cohort study Patients with CD or UC were recruited from the Danish National Patient Register (2000–2017). Information on antidepressant use and proxy measures of disease activity (healthcare and drug utilization) was extracted. Disease activity rates by antidepressant use adjusted for confounders were estimated with Poisson regression. The analyses were performed stratified by IBD subtype and type of antidepressants.	As surrogate markers of disease relapse the primary outcomes were defined as either (1) hospitalization with IBD as the primary diagnosis; (2) surgery associated with IBD or (3) step-up medication in terms of a redeemed prescription of corticosteroids.	N/A	The antidepressant group had a significantly lower relapse rate (IRR, 0.85; 95% CI, 0.81–0.90) compared with the other patients. The association was more pronounced in patients with CD compared with UC patients. Patients with no prior use of antidepressants before IBD onset had a favorable influence on the disease course when exposed to antidepressants compared with nonusers.	N/A	17 years (2000–2017)
Chojnacki et al. (2011) [33]	Tianeptine	60 UC patients in remission.	Clinical trial: Patients were divided in 2 groups of 30 subjects: the first (group 1) received aminosalicylates (2.0 g daily) and tianeptine (3 × 12.5 mg) and the second (group 2) received placebo. Symptoms of anxiety, depression and IBD clinical activity were assessed and compared between the two groups every 3 months for 1 year.	MCDAI, hemoglobin and CRP were evaluated to assess disease activity.	Anxiety and depression were assessed, respectively, with Hamilton Anxiety Rating Scale-HARS and Back Depression Inventory-BDI.	At 12 months, a significant decrease in disease activity index (respectively, 3.05 ± 1.36 and 4.65 ± 1.69), insignificantly lower level of CRP (7.00 5.65 and 9.41 ± 10.12) and higher level of hemoglobin (11.93 ± 0.83 and 11.0 ± 0.70) were observed in the tianeptine group compared with placebo group.	After 12 months, significant decreases in anxiety (from 20.35 ± 4.03 to 12.65 ± 3.78 points) and depression (from 19.95 ± 4.49 points to 9.60 ± 2.76 points) were observed in the tianeptine group compared with placebo group.	12 months
Hall et al. (2018) [25]	59.3% SSRI 31.5% TCA 3.7% SNRI 3.7% both SNRI and TCA 1.9% trazodone	331 patients with an established radiological, endoscopic or histological diagnosis of CD or UC.	Longitudinal cohort study: Cohort 1: 54 patients treated with antidepressant at baseline. Cohort 2: 277 patients not treated with antidepressant Participants were followed for a minimum period of 2 years to assess the occurrence of 4 clinical endpoints.	Longitudinal disease activity was assessed with 4 outcomes: - disease flares or need for glucocorticoids; - escalation of medical therapy; - hospitalization; - intestinal resection	Anxiety and depression data were collected at baseline using the hospital anxiety and depression scale (HADS). Somatization data were collected at baseline using the patient health questionnaire-15 (PHQ-15).	During longitudinal follow-up, there was a trend towards lower rates of any of the four endpoints of IBD activity in patients with abnormal anxiety scores at baseline and who were receiving an antidepressant (42.3% versus 64.6%, *p* = 0.05). Based on univariate Cox regression analysis, there was a trend towards lower rates of escalation of medical therapy among patients receiving antidepressants at baseline (HR = 0.59; 95% CI 0.35–1.00, *p* = 0.05).	N/A	2-year follow-up

Crohn’s disease (CD); Ulcerative colitis (UC); Inflammatory bowel disease (IBD); Crohn’s Disease Activity Index (CDAI); Mayo Clinic Disease Activity Index (MCDAI); C-reactive protein (CRP); Erythrocyte Sedimentation Rate (ESR); albumin (ALB); white blood cells (WBC); selective serotonin reuptake inhibitors (SSRIs); serotonin noradrenergic reuptake inhibitors (SNRIs); Tricyclic Antidepressant (TCA); Inflammatory Bowel Disease Questionnaire (IBDQ); Hospital Anxiety and Depression Scale (HADS), World Health Organisation Quality of Life questionnaire (WHOQoL); Not Available (N/A); Quality of Life (QoL); Lichtiger Colitis Activity Index (LCAI); Short Form-36 (SF-36) and Arizona Sexual Experience Scale (ASEX); Hazard Ratio (HR).

**Table 2:** Included studies on antiepileptics for gastrointestinal outcomes in IBD patients.

				Outcomes		Results		
Author (Year), Ref.	Drug	Study Population	Study Design	IBD	Psychiatric Condition	IBD	Psychiatric Condition	Follow-Up
Crockett et al. (2014), [38]	Topiramate	1733 IBD subjects CD = 955 UC = 755 IBD-U = 23	Retrospective cohort study Study group (n = 775) treated with Topiramate (≤50 mg/day and ≥100 mg/day dose) Control group (n = 958) treated with comparator drugs (other anticonvulsant and anti-migraine medications)	Primary: new prescription for an oral steroid (≥14 days). Secondary: initiation of biologic agents, abdominal surgery, and hospitalization	N/A	Topiramate use was not associated with the primary outcome of steroid prescriptions (HR 1.14, 95% CI 0.74, 1.73). Results did not differ significantly by IBD subtype. There is no difference between the cohorts respect to post-exposure initiation of biologic agents (HR 0.93, 95% CI 0.35, 2.52), abdominal surgery (HR 1.22, 95% CI 0.70, 2.12), or hospitalization (HR 0.78, 95% CI 0.49, 1.26).	N/A	Median follow-up of 2.8 months
D’Onofrio et al. (2024) [39]	Pregbalin	1 CD patient	Case Report	Assessment of HBI, SES-CD and CRP after therapy with pregabalin	N/A	After 2 months of treatment: HBI dropped from 9 to 4 and CRP normalized. After 6 months of treatment SES-CD dropped from 17 to 6.	N/A	6 months

Crohn’s disease (CD); Ulcerative colitis (UC); IBD-unclassified (IBD-U); Inflammatory bowel disease (IBD); Harvey Bradshaw index (HBI); Simple Endoscopic Score for Crohn’s Disease (SES-CD); Not Available (N/A); C-reactive protein (CRP); Hazard Ratio (HR).

**Table 3:** Included study on mood stabilizers for gastrointestinal outcomes in IBD patients.

					Outcomes		Results		
Author (Year), Ref.	Drug	Disease Type	Study Population	Study Design	IBD	Psychiatric Condition	IBD	Psychiatric Condition	Follow-Up
Zisook et al. (1972) [42]	Lithium carbonate	UC	Case report 67 years old man with cyclic manic-depressive psychosis and UC	Administration of lithium carbonate in the manic phase of the patient with also frequent, bloody stools with recent endoscopic findings of “severe, active ulcerative colitis, already on salicosulfapiridine (azulfadine) therapy”.	N/A	N/A	After 5 days of therapy evidence of marked improvement in frequency of evacuations and bleeding. After two months endoscopic evidence of revealed marked improvement with only a few small bleeding points.	No more overtly psychotic	16 months

Ulcerative colitis (UC); Not Available (N/A).

**Table 4:** Included studies on other psychiatric medications for gastrointestinal outcomes in IBD patients.

				Outcomes		Results		
Author (Year), Ref.	Drug	Study Population	Study Design	IBD	Psychiatric Condition	IBD	Psychiatric Condition	Follow-Up
Furlan et al. (2006) [48]	Clonidine	23 UC patients 20 controls	Clinical Trial First part: UC patients were compared with 20 healthy controls regarding the neural autonomic profile. Secondo part: a subgroup of 16 patients randomly assigned to 8-wk transdermal clonidine (15 mg/weeks, 9 subjects), or placebo (7 patients).	DAI and endoscopic pattern before and after clonidine/placebo.	N/A	Changes in the autonomic profile after clonidine were associated with a reduction in the DAI score. Normalization of the autonomic profile with clonidine was accompanied by an improvement of the disease and a general increase in sympathetic activity characterized active UC.	N/A	8 weeks
Lechin et al. (1985) [47]	Clonidine	45 UC patients with at least one year of disease duration and severe activity (10 or more bloody stools per day for 15 or more days, not treated with steroids or sulfasalazine in the previous three months)	Double-blind Clinical Trial Prednisone (n = 15) 20 mg tid. Sulfasalazine (n = 15) 1.5 mg tid. Clonidine (n = 15) 0.3 mg tid. Each group was treated for three six-week periods, separated by two six-week placebo periods.	Assessment of rating scales for clinical, endoscopic, histologic, and radiologic changes. biochemical parameters. Distal colon motility changes.	N/A	Clonidine and prednisone were effective in treating idiopathic UC. Both drugs were more effective than sulfasalazine. Furthermore, clonidine potentiated prednisone and sulfasalazine effects.	N/A	30 weeks followed by an open evaluation of at least one year
Lie et al. (2018) [43]	Naltrexone	47 IBD patients (CD = 28 UC = 19)	Clinical trial: participants were steroid dependent or steroid refractory with clinical active disease at initiation of LDN (4.5 mg Naltrexone once daily) therapy. 87.2% previously received at least one anti-TNFα agent. 40.4% had been treated with two anti-TNFα agent.	Clinical response = self-assessed disease activity decreased within the first 4 weeks of therapy and lasted for at least 4 weeks. Endoscopic findings were scored from 0 to 3, (no inflammation to severe inflammation) Consecutive endoscopies were performed at baseline and at 12 weeks or at time of relapse, whichever occurred earlier.	N/A	- 35 (74.5%) achieved a clinical response. - No statistically significant difference between CD and UC - Clinical remission had a significantly greater improvement in endoscopic score compared to not clinical response (median change −1.5, range −2 to 0 versus 1.0, range 0–2, p = 0.005). - 6 patients with response and 6 patients with remission had consecutive endoscopies evaluation and no significant difference was observed in baseline endoscopic score for response and remission.	N/A	12 weeks
Paulides et al. (2022) [49]	Naltrexone	122 patients with mild to moderately active CD, defined by endoscopy SES-CD of 3–15.	Randomized, double-blinded, placebo-controlled multicenter clinical trial: - 61 patients = LDN 4.5 mg once daily - 61 patients = placebo, for 12 weeks. - After 12 weeks patients are invited to an open label exploration extension study until week 52.	Primary objective defined as SES-CD ≤2 and an ulcerated surface subscore ≤1 in all five segments at week 12. Secondary: - Steroid free clinical remission (HBI score of ≤4 complete tapering of systemic corticosteroids) - Clinical Response (decrease in HBI of ≥3 points compared with baseline) - Endoscopic response (reduction in SES-CD score by ≥50% vs. baseline at week 12). - Changes in laboratory inflammation (CRP and FC) at week 12, 24 and 52. - Steroid free clinical remission at week 52. - Endoscopic remission and response at week 52.	- Quality of life, via the SIBDQ and EQ5D. - Fatigue, ia the FACIT-F and MFI. - Anxiety, depression and sleep disturbance, via the PROMIS NIH.	ongoing study	ongoing study	52 weeks
Smith et al. (2011) [44]	Naltrexone	40 CD patients with moderate to severe activity (CDAI of ≥220)	Randomized double-blind placebo-controlled study. - 18 patients = naltrexone 4.5 mg - 16 patients = placebo - 32 patients = naltrexone 4.5 mg the open-labeled extend study (additional 12 weeks)	Primary outcome: clinical response (70-point decline in CDAI scores from baseline values at 12 weeks). Secondary outcome: - endoscopic healing with colonoscopy and biopsies. - endoscopic response (5-point decline in CDEIS)	N/A	- 88% of active arms had at least a 70-point decline in CDAI scores compared to 40% of placebo (*p* = 0.009). - 78% of active arm exhibited an endoscopic response compared to 28% response in placebo-treated controls (*p* = 0.008), - 33% of active arm achieved remission with a CDEIS compared to 8% of placebo. Open labeled: - patients who continued naltrexone therapy had a further 75- point decline in CDAI scores (*p* < 0.01) -24-week score was also significant compared to baseline (*p* < 0.0001).	N/A	12 weeks (+additional 12 weeks)
Smith et al. (2007) [45]	Naltrexone	17 = CD treated with 4.5 mg naltrexone/day.	Open-labeled pilot prospective trial including CD patients with activity index (CDAI) score of 220–450, with no active biological therapy treated with naltrexone.	CDAI scores were assessed pretreatment, every 4 weeks on therapy and 4 weeks after completion of the study drug.	IBDQ and the short-form (SF-36) quality of life surveys	CDAI scores decreased significantly (*p* = 0.01) with LDN, from baseline to 4 weeks after completing therapy. 89% of response (*p* < 0.001) 67% of remission.	Improvement in both quality-of-life surveys with LDN compared with baseline	12 weeks
Smith et al. (2013) [46]	Naltrexone	CD = 12 (pediatric patients mean age of 12.3 years) moderate to severe Crohn’s disease (PCDAI of >30), not in therapy with steroids greater than 10 mg daily, anti-TNFα agents	Pilot clinical trial Study group: 6 CD treated with naltrexone (0.1 mg/kg) Placebo: 6 CD 11 CD = open-labeled treatment with 8 additional weeks of naltrexone.	Primary outcome: safety and tolerability compared to placebo. secondary outcome: clinical response with 10-point decline in PCDAI scores compared to baseline/pretreatment values.	Quality of life was monitored by the Impact III survey.	- naltrexone was well tolerated without any serious adverse events -PCDAI scores significantly decreased from pretreatment values (34.2 ± 3.3) with an 8-week course of naltrexone therapy (*p* = 0.005). - 25% of those treated with naltrexone were considered in remission (score ≤10) and 67% had improved with mild disease activity (decrease in PCDAI score by at least 10 points) at the end of the study.	Systemic and social quality of life improved with naltrexone treatment (*p* = 0.035).	16 weeks

Crohn’s disease (CD); Ulcerative colitis (UC); Crohn’s Disease Activity Index (CDAI); Disease Activity Index (DAI); Harvey Bradshaw index (HBI); Simple Endoscopic Score for Crohn’s Disease (SES-CD); Not Available (N/A); C- reactive protein (CRP); Fecal calprotectin (FC); Inflammatory Bowel Disease Questionnaire (IBDQ); Hospital Anxiety and Depression Scale (HADS), World Health Organization Quality of Life questionnaire (WHOQoL); Not Available (N/A); Quality of Life (QoL); Short Inflammatory Bowel Disease Questionnaire (SIBDQ); Ter in die (tid); CDEIS (Crohn’s Disease Endoscopic Index of Severity); Pediatric Crohn’s Disease Activity Index (PCDAI); 5-level EQ-5D version (EQ-5D-5L); Functional Assessment Chronic Illness Therapy (Fatigue) (FACIT-F); Multi-Dimensional Fatigue Inventory (MFI); Patient-Reported Outcomes Measurement Information System (PROMIS); Low dose Naltrexone (LDN); Tumor Necrosis Factor (TNF).

## Data Availability

This systematic review is based on publicly available data from previously published studies. All data used in this review are accessible through the cited articles and their respective publishers. No new data were generated for this study.

## Supplementary Materials

The supporting information can be downloaded at: https://www.mdpi.com/article/10.3390/ijms26136514/s1. References [104,105,106,107,108,109,110,111,112,113,114,115,116,117,118,119,120] are cited in the supplementary materials.
