# Supplementary material for: Psychopharmacological Therapy Positively Modulates Disease Activity in Inflammatory Bowel Disease: A Systematic Review
Source: Int J Mol Sci. 2025 Jul 6;26(13):6514. doi: 10.3390/ijms26136514 (PMC12249696; doi:10.3390/ijms26136514)
Supplement: Supplementary file 1 [file ijms-26-06514-s001.zip › ijms-3683353-supplementary.pdf]

Supplementary Table S1: Instruments used in the eligible studies.

Assessment Tool	Outcome Assessed	Number of Studies	Positive Effect (n)	No Effect (n)
**ASEX**	*Sexual Experience*	1	1<br>Yanartas et al., 2016 (27)	0
**BDI**	*Depressive symptoms*	1	1<br>Chojnacki et al., 2011 (33)	0
**CDAI**	*Severity of CD*	7	6<br>Yanartas et al., 2016 (27)<br>Kane et al., 2003 (34)<br>Kast and Altschuler et al., 2001 (54)<br>Liang et al., 2022 (31)<br>Smith et al., 2011 (43)<br>Smith et al., 2007 (44)	1<br>Mikocka-Walus et al., 2017 (29)
**General likert scale for symptoms or endoscopic features**	*Severity of IBD*	2	2<br>Iskandar et al., 2014 (32)<br>Liet et al., 2018 (101)	0
**HADS**	*Anxiety and Depression*	4	3<br>Daghaghzadeh et al., 2015 (30)<br>Yanartas et al., 2016 (27)<br>Liang et al., 2022 (31)	1<br>Mikocka-Walus et al., 2017 (29)
**HBI**	*Severity of CD*	1	1<br>D'Onofrio et al., 2024 (38)	0
**HARS**	*Anxiety symptoms*	1	1<br>Chojnacki et al., 2011 (33)	0
**IBDQ**	*Quality of life in IBD patients*	2	2<br>Liang et al., 2022 (31)<br>Smith et al., 2007 (44)	0
**IMPACT-III**	*Quality of life*	1	1<br>Smith et al., 2013 (45)	0
**LCAI**	*Severity of UC*	1	1<br>Daghaghzadeh et al., 2015 (30)	0
**Mayo score**	*Severity of UC*	2	2<br>Liang et al., 2022 (31)<br>Furlan et al., 2006 (47)	0
**MCDAI**	*Disease activity*	1	1<br>Chojnacki et al., 2011 (33)	0
**mMS**	*Severity of UC*	1	0	1<br>Yanartas et al., 2016 (27)
**No assessment tool used (or only at baseline*)**	8<br>Goodhand et al., 2012 (28)<br>Kast et al., 1998 (35)<br>Kristensen et al., 2019 (26)<br>Hall et al., 2018 (25)*<br>Crockett et al., 2014 (37)<br>Zisook et al., 1972 (41)<br>Lechin et al., 1985 (46)<br>Paulides et al., 2022 (42)*			
**PCDAI**	*Severity of CD in pediatric population*	1	1<br>Smith et al., 2013 (45)	0
**SES-CD**	*Severity of endoscopic findings in CD*	2	1<br>D'Onofrio et al., 2024 (38)	1<br>Liang et al., 2022 (31)
**SF-36**	*Health-related quality of life*	2	2<br>Yanartas et al., 2016 (27)<br>Smith et al., 2007 (44)	0
**UCEIS**	*Severity of endoscopic findings in UC*	1	0	1<br>Liang et al., 2022 (31)
**WHOQoL**	*Quality of life*	1	0	1<br>Mikocka-Walus et al., 2017 (29)
**WHOQoL-BREF**	*Quality of life*	1	1<br>Daghaghzadeh et al., 2015 (30)	0

Abbreviations. Arizona Sexual Experience Scale (ASEX); Beck Depression Inventory (BDI); CD (Crohn's disease); Crohn's disease activity index (CDAI); Hospital Anxiety and Depression Scale (HADS); Harvey Bradshaw index (HBI); Hamilton Anxiety Rating Scale (HARS); Inflammatory Bowel Disease Questionnaire (IBDQ); Lichtiger Colitis Activity Index (LCAI); Microscopic Colitis Disease Activity Index (MCDAI); Modified Mayo Score (mMS); Pediatric Crohn's Disease Activity Index (PCDAI); Short form 36 (SF-36); Simple Endoscopic Score for Crohn's Disease (SES-CD); UC (Ulcerative Colitis); Ulcerative Colitis Endoscopic Index of Severity (UCEIS); World Health Organization Quality of Life – BREF (WHOQOL-BREF). Note: The total does not match the number of studies. Some used multiple tools. For example, in studies with both UC and CD patients.

Supplementary Table S2. Characteristics of included medications.

Drug class / Drug	Mechanism of action	FDA-approved indications	Common GI-specific side effects	Authors' Comments
**Antidepressants**				
**SSRIs**				
**Citalopram** [104]	Boosts neurotransmitter serotonin.	Depression.	Decreased appetite, nausea, diarrhea, constipation, dry mouth.	Gastrointestinal side effects typically show improvement within one week. In certain cases, H1 blockade may enhance anxiolytic effects, as well as appetite and/or weight.
**Escitalopram** [105]	Boosts neurotransmitter serotonin.	Major depressive disorder, generalized anxiety disorder.	Decreased appetite, nausea, diarrhea, constipation, dry mouth.	Blocking the serotonin transporter (SERT) may amplify antidepressant effects. However, QTc prolongation necessitates careful monitoring and management.
**Fluvoxamine** [106]	Boosts neurotransmitter serotonin.	Obsessive-compulsive disorder, social anxiety disorder.	Decreased appetite, nausea, diarrhea, constipation, dry mouth.	The presence of comorbid obsessive-compulsive disorder and psychotic features during a Major Depressive Episode could signify a unique treatment focus.
**Paroxetine** [107]	Boosts neurotransmitter serotonin.	Major depressive disorder, obsessive-compulsive disorder, panic disorder, social anxiety disorder, posttraumatic stress disorder, generalized anxiety disorder, premenstrual dysphoric disorder, vasomotor symptoms.	Decreased appetite, nausea, diarrhea, constipation, dry mouth.	Strengths: Broad spectrum of indications, with particular efficacy in managing anxiety comorbidity.<br>Weaknesses: Initial gastrointestinal side effects, typically ameliorating within the first two weeks; inhibition of CYP2D6; propensity for weight gain; notable affinity for the M1 receptor, potentially leading to anticholinergic effects; increased impulsivity observed in children and adolescents, correlating with higher suicidal risk.
**Sertraline** [108]	Boosts neurotransmitter serotonin.	Major depressive disorder, premenstrual dysphoric disorder, panic disorder, posttraumatic stress disorder, social anxiety disorder, obsessive-compulsive disorder.	Decreased appetite, nausea, diarrhea, constipation, dry mouth.	Comorbidity of obsessive-compulsive disorder, posttraumatic stress disorder, and anxiety presents a focused treatment opportunity. Additionally, the medication shows improved tolerability in children and adolescents.
**SNRIs**				
**Duloxetine** [109]	Boosts neurotransmitters serotonin, norepinephrine/ noradrenaline and dopamine.	Major depressive disorder, diabetic peripheral neuropathic pain, fibromyalgia, generalized anxiety disorder, chronic musculoskeletal pain.	Nausea, diarrhea, decreased appetite, dry mouth, constipation.	Chronic comorbidities such as anxiety, pain, and somatic symptoms are a key treatment focus.<br>Caution: Regular monitoring of heart rate, systolic, and diastolic blood pressure is advised.
**Venlafaxine** [110]	Boosts neurotransmitters serotonin, norepinephrine/ noradrenaline and dopamine.	Depression, generalized anxiety disorder, social anxiety disorder, panic disorder.	Nausea, diarrhea, decreased appetite.	The medication exhibits dosage-dependent aminergic properties, with increased dosage correlating with greater aminergic activity. Improved tolerability is observed with devices that modulate release.
**DA/NA RIs, NaSSA and MT agonists**				
**Bupropion** [111]	Boosts neurotransmitters norepinephrine/ noradrenaline and dopamine.	Major depressive disorder, seasonal affective disorder, nicotine addiction.	Dry mouth, constipation, nausea, weight loss, anorexia, abdominal pain.	Enhanced gastrointestinal tolerability is noted in comparison to serotonergic medications. Overweight/obesity and atypical symptoms are highlighted as specific treatment targets. Cautionary measures include monitoring heart rate, systolic and diastolic blood pressure, and being mindful of the potential for lowering the seizure threshold.
**Mirtazapine** [112]	Boosts neurotransmitters serotonin and norepinephrine/ noradrenaline.	Major depressive disorder.	Dry mouth, constipation, increased appetite, weight gain.	The medication's noradrenergic and targeted serotonergic properties, attributed to the blockade of post-synaptic receptors 5-HT2 and 5-HT3, potentially enhance gastrointestinal tolerability. Melancholic features such as insomnia and anorexia are identified as specific treatment targets. However, caution is advised regarding sedation and weight gain.
**Agomelatine** [113]	Synergistic melatonergic and serotonergic action that increase norepinephrine and dopamine neurotransmission in the prefrontal cortex.	No FDA approved indications, but commonly prescribed for depression and generalized anxiety disorder.	Nausea (less common: diarrhea, constipation, upper abdominal pain, vomiting, increase of transaminase levels).	Gastrointestinal tolerability is generally favorable.<br>Caution: Monitor transaminase levels closely.
**Tricyclic Antidepressants** [114]	Boosts neurotransmitters serotonin (not all) and norepinephrine/ noradrenaline.	Depression (all of them), some for mitigation of opioid withdrawal symptoms to facilitate abrupt opioid discontinuation, some for obsessive-compulsive disorder.	Constipation, increased appetite, dry mouth, nausea, diarrhea, unusual taste in mouth, weight gain.	The medication demonstrates effective antidepressant properties, though with reduced tolerability and safety, primarily due to anticholinergic, antihistaminic, and adrenolytic side effects. Caution is advised, particularly in vulnerable individuals, regarding the risk of arrhythmia resulting from the blockade of cardiac sodium channels.
**MAO-Inhibitors** [115]	Boosts noradrenergic, serotonergic, and dopaminergic neurotransmission. Some block tyramine's metabolism in the gut.	Depression. Some for Parkinson's disease or symptomatic parkinsonism (adjunctive treatment).	Constipation, dry mouth, nausea, change in appetite, weight gain. Some can cause diarrhea.	The medication exhibits strong antidepressant efficacy, yet its tolerability and safety profile is less favorable. Caution is warranted, necessitating vigilance in monitoring for potential drug interactions and adhering to dietary restrictions. Additionally, there is a risk of cardiovascular events and adverse hypertensive reactions that require attention.
**Mood stabilizers and opioid modulators**				
**Topiramate** [116]	Blocks voltage-sensitive sodium channels by an unknown mechanism.	Partial onset seizures, primary generalized tonic-clonic seizures, seizures associated with Lennox-Gastaut syndrome, migraine prophylaxis, chronic weight management.	Nausea, appetite loss, weight loss, and altered taste perception.	Comorbidities such as overweight/obesity may benefit from appetite suppression. However, the medication's mood stabilizing properties are not firmly established.
**Lithium** [117]	Unknown and complex mechanism of action.	Maintenance treatment for manic-depressive patients with a	Diarrhea, nausea, weight gain.	The medication demonstrates excellent mood stabilizing properties and has the potential to augment antidepressant efficacy. It is particularly valuable in

		history of mania, bipolar maintenance.		suicide prevention efforts. However, caution is necessary due to its narrow therapeutic index, necessitating close monitoring for renal toxicity, cardiovascular, and endocrine effects.
**Naltrexone** [118]	Block mu opioid receptors, preventing exogenous opioids from binding there and thus preventing the pleasurable effects of opioid consumption. Reduces alcohol consumption through modulation of opioid systems, thereby reducing the reinforcing effects of alcohol.	Alcohol dependence, blockade of effects of exogenously administered opioids, prevention of relapse to opioid dependence.	Nausea, vomiting, decreased appetite.	The medication's anti-craving properties can be advantageous for dependent individuals. Comorbidities like overweight/obesity may benefit from appetite suppression, particularly when combined with bupropion. However, caution is advised, necessitating regular monitoring of liver function.
**$GABA_A$ enhancers**				
**Benzodiazepines** [119]	They bind to benzodiazepine receptors enhancing the inhibitory effect of GABA.	Mainly for anxiety, insomnia, and neurological conditions.	Some can cause dry mouth. Some can cause hypersalivation. Midazolam can cause nausea and vomiting.	The medication offers rapid relief for acute anxiety and insomnia, making it suitable for short-term use. However, caution is warranted due to the risk of tolerance and dependence, as well as potential cognitive, depressogenic, and disinhibiting side effects.
**Z-drugs** [120]	They bind to a subtype of the benzodiazepine receptor enhancing GABA inhibitory actions.	Insomnia.	Some can cause dry mouth, loss of appetite (or decrease appetite), constipation, bitter taste. Some can cause diarrhea and nausea.	Effective for short-term management of insomnia. Caution: Risk of tolerance and dependence; contraindicated in individuals with sleep apnea.

Abbreviations: $GABA_A$, gamma-aminobutyric acid receptor type A; MAO, monoamine oxidase; NaSSA, Noradrenaline-serotonin specific antagonists; SNRIs, Serotonin and norepinephrine (noradrenaline) reuptake inhibitors; SSRIs, Selective serotonin reuptake inhibitors; Z-drugs, nonbenzodiazepine $GABA_A$ activators.
